# Adapting Global Dietary Recommendation Indices to Assess Retail Food Environment Quality: Spatial Insights from Rural and Urban Kenya

**DOI:** 10.1016/j.cdnut.2026.107685

**Published:** 2026-04-03

**Authors:** Nicanor Obiero Odongo, Tosin Harold Akingbemisilu, Irmgard Jordan, Fifali Sam Ulrich Bodjrenou, Juliana Kiio, Ramona Teuber, Céline Termote

**Affiliations:** 1 Food Environment and Consumer Behavior, International Center for Tropical Agriculture (CIAT), Nairobi, Kenya; 2 Institute of Agricultural Policy and Market Research, Justus Liebig University Giessen, Giessen, Germany; 3 Food Environment and Consumer Behavior, Bioversity International, Nairobi, Kenya; 4 Food Environment and Consumer Behavior, CIAT, Cotonou, Benin; 5 Department of Food, Nutrition and Dietetics, Kenyatta University, Nairobi, Kenya; 6 Center for Sustainable Food Systems, Justus Liebig University Giessen, Giessen, Germany

**Keywords:** food environment quality, spatial autocorrelation, healthy food environment, unhealthy food environment, food environment indices

## Abstract

**Background:**

Rapid nutrition transitions in low- and middle-income countries are increasingly shaped by retail food environments, influencing dietary choices and contributing to rising burdens of nutrition-related noncommunicable diseases (NCDs). However, standardized and scalable metrics for characterizing retail food environment quality in relation to dietary recommendations and NCD risk remain limited.

**Objectives:**

This study aimed to adapt Global Dietary Recommendation (GDR) indices to assess retail food environment quality by quantifying the availability of food groups associated with NCD risk and protection.

**Methods:**

A cross-sectional market survey was conducted among food retailers in one rural and one urban administrative ward in Kenya. Data were collected on food groups sold, retail outlet typology, retailer gender, and geographic coordinates. Three indices were constructed: the Healthy Retail Food Environment Score (HRFES), the Unhealthy Retail Food Environment Score (URFES), and the Retail Food Environment Quality Index (RFEQI). Fixed-effect regression models examined associations between retail typology and index scores, whereas spatial autocorrelation analyses identified clustering patterns of healthy and unhealthy food availability.

**Results:**

Stall and tabletop retailers were strongly associated with higher HRFES [incidence rate ratio (IRR): 3.15; 95% confidence interval (CI): 2.61, 3.85; *P* < 0.001] and higher overall food environment quality [RFEQI; IRR: 1.19; 95% CI: 1.12, 1.27; *P* < 0.001]. In contrast, supermarkets were most strongly associated with higher URFES [IRR: 11.30; 95% CI: 6.96, 18.5; *P* < 0.001] and substantially lower RFEQI [IRR: 0.62; 95% CI: 0.41, 0.88; *P* < 0.05]. Spatial analyses showed more pronounced clustering of unhealthy food groups than healthy food groups across both rural and urban settings.

**Conclusions:**

Adapted GDR-based indices offer a robust and policy-relevant approach for assessing retail food environment quality and its spatial distribution. These tools support monitoring, cross-context comparisons, and targeted interventions to improve diet quality and reduce NCD risk in rapidly transforming food systems.

## Introduction

In sub-Saharan Africa (SSA), there is growing concern about a rapid nutrition transition that affects both rural and urban populations [1]. Diets are shifting away from traditional foods toward convenient ultraprocessed foods, which are often high in sugar, salt, *trans* fats, and saturated fats [[2], [3], [4], [5]]. This transition is fueling a rising burden of nutrition-related noncommunicable diseases (NCDs) [1,6]. Remarkably, in many low- and middle-income countries (LMICs), the prevalence of NCDs is now increasing more rapidly in rural areas than in urban centers [7]. The growing nutrition-related NCD burden has been linked to increased reliance on retail food environments among consumers across both rural and urban areas. The retail outlets increasingly sell processed foods, thereby heightening consumers’ exposure to diet-related risks [[8], [9], [10]]. Although national strategies increasingly recognize the need to address diet-related NCD risks, the implementation of food environment policy measures (e.g., marketing restrictions, interpretive front-of-pack labeling, fiscal measures targeted at sugar, salt and ultraprocessed foods) remains ineffective, diluted, and fragmented, a major barrier to sustainable food systems that support healthy food, especially within informal retail markets [11,12]. These strategies are in line with Healthy Food Environment Policy Index country scorecards and priority recommendations for action in Kenya [13]. Recent developments—such as the release of the Kenya Nutrient Profile Model in 2025 [14], intended to guide front-of-pack nutrition labeling and related interventions—indicate growing policy momentum, although these measures are still in the early stages of rollout. Therefore, there is a need to better understand the quality of local retail food environments in relation to NCD risk to inform effective policy interventions.

Food environments have been defined as “the space within the food system where consumers make decisions about what to acquire and consume” [[15], [16], [17]]. Turner et al. [5] (2018) food environment framework distinguishes two domains that mediate food choices: an external domain, which refers to structural factors operating outside the individual, and a personal domain, which refers to individual-level characteristics and circumstances that shape how people interact with the food environment. The external domain comprises food availability, which precedes price, retail properties, and marketing regulations [5]. Once food is available, personal domain factors such as accessibility, affordability, convenience, and desirability shape actual food choices. Retail characteristics such as retail typology, location, and retailers’ gender further influence the range of food groups offered at the retail level [18,19]. Examining these characteristics is therefore essential to understanding how retail-level dynamics shape food environment quality and, in turn, may influence consumer diets.

Despite recognition of the central role of retail food environments in shaping diet and health outcomes, quantitative measures to assess and monitor retail food environment quality with regard to NCD-risk contribution remain limited. Existing indicators tend to focus on dietary diversity scores, which are designed primarily for nutrient adequacy [3,[20], [21], [22], [23]]. Diet diversity scores do not effectively capture whether the retail food environment promotes healthy or unhealthy diets [24]. Similarly, the NOVA classification, which categorizes food as unprocessed, culinary processed, or ultraprocessed, has been widely used to characterize food environment quality [23,[25], [26], [27]], but faces limitations; it does not account for nutrient composition to inform whether a product is healthy or not [28] and often lacks local nuances and conditions [29]. There is therefore a need to develop validated indices that can more fully consider nutrient-dense food availability and directly capture retail food environment quality in relation to obesity and NCD risks [30,31].

Other studies have employed dietary assessment indices, such as the Healthy Eating Index and the Global Diet Quality Score, to characterize the retail food environment [29,32]. However, these indices require data on food quantities, which are more feasible in high-income countries where formal retail systems predominate. In LMICs, where food retail systems are predominantly informal, applying such indices is more challenging as collecting data on food quantities at the retail level is logistically difficult and costly. By contrast, the “Access to Nutrition Initiative” adopted a different approach by applying nutrient profile analysis of selected packaged foods to assess their healthiness. Findings indicated that in Tanzania and Kenya, only 25% and 33% of products analyzed, respectively, met the healthy threshold under the Health Star Rating system [33,34]. Although nutrient profiling offers more accurate insights into retail food environment quality, it remains expensive and time-consuming. The Nutrition-Sensitive Food Environment Index also employed a healthy and an unhealthy food count to characterize the retail food environment. However, this approach did not assess any association with health outcomes, such as diet-related NCDs [20]. Other studies have reported that most food environment indices that exist were developed in the context of high-income countries and may fail to accurately capture the context in LMICs [35]. Collectively, these challenges underscore the need for simple, low-cost, yet reliable tools to assess retail food environment quality as either healthy or unhealthy, particularly in LMICs.

Recent advances, such as the diet quality questionnaire (DQQ) used for assessing the global diet quality score [36], provide a promising foundation for retail food environment quality assessment. The DQQ identifies nine food groups recommended for protection against NCDs and eight food groups associated with increased NCD risk in line with WHO recommendations [37]. Researchers need validated indices to characterize retail food environment quality in relation to NCD risks. They are essential for monitoring food environments across different geographical locations and for guiding policies toward healthier, sustainable retail systems [30]. Importantly, spatial approaches can complement these assessments by identifying geographic patterns, including unhealthy food hot spots, where these foods dominate, and cold spots of healthy options, thereby informing targeted interventions. Although previous studies have examined food group distributions and household proximity [3,18,21,29], few have systematically linked retail food availability with spatial clustering patterns that could guide location-specific policy action.

This study addresses two main gaps in characterizing retail food environment quality in LMICs. First, it adapts validated dietary assessment indices to quantitatively assess the quality of the retail food environment in terms of the availability of healthy and unhealthy foods. Second, it develops a methodological approach for identifying locations that are hot or cold spots for either unhealthy or healthy food categories. By operationalizing Global Dietary Recommendation (GDR) indicators at the retail outlet level and embedding them within a spatial analytical framework, we introduce a novel approach that bridges global dietary guidelines with food environment assessment in LMIC contexts. Addressing these gaps will yield innovative retail food environment monitoring tools that could be applied for various purposes: *1*) comparing retail food environment quality across different regions or countries; *2*) classifying retail food environment as hot or cold spots for healthy or unhealthy food categories; *3*) informing targeted interventions suitable for local retail food environments identified as requiring improvements; and *4*) tracking changes in retail food environment quality over time in different regions to assess the effectiveness of implemented interventions. This will support transforming the retail food environment to ensure that healthy food options are available.

## Methods

### Study area and design

**FIGURE 1:**
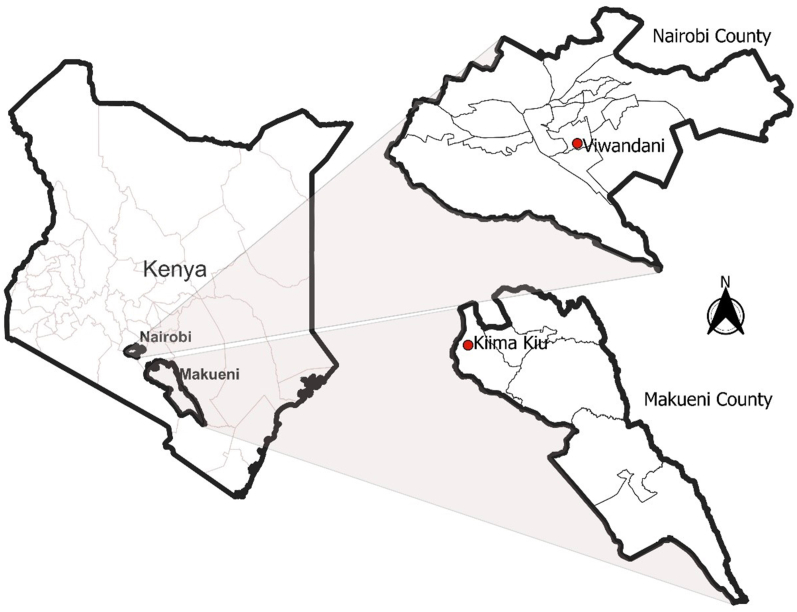
Map of the study areas in relation to their location in Kenya.

This study employed a cross-sectional market survey and an observational audit of available food items, in which all food retail outlets within the study sites were geocoded [20,36]. Data collection took place in two contrasting settings in Kenya: Kiima Kiu ward in Makueni County, representing a rural retail food environment, and Viwandani ward, an informal settlement in Nairobi County, representing a low-income urban retail food environment (Figure 1). The study forms part of a broader research initiative aimed at strengthening linkages between urban markets and smallholder farmers practicing agroecological principles. Study sites were purposively selected in consultation with community leaders and project partners, based on the dual criteria of their relevance to ongoing agroecological interventions and the high burden of NCDs in the respective population.

### Ethical approval

Before the study was conducted, ethical approval was obtained from both the institutional review board of the Alliance of Bioversity International and CIAT (International Center for Tropical Agriculture) (2023-IRB22) and the Amref Ethics and Scientific Review Committee (ESRC P1451/2023). Additional approval was granted by the Kenya National Commission for Science, Technology, and Innovation (NACOSTI/P/23/28871) to ensure compliance with national regulations. Written consent was obtained from retailers before the assessment was conducted.

### Data collection

The data collection tool was adapted from previously validated retail food environment assessment tools [20]. To enhance content validity and contextual relevance, the tool was refined to encompass the full range of food items available at retail outlets―the tool, as used in this study, is published in a separate paper [38]. The data collection tool was pretested outside the study sites in a ward with similar socioeconomic characteristics. Feedback from the pretest informed revisions to improve the tool’s clarity, comprehensiveness, and usability. Once refined, the tool was digitized and implemented through the Alliance FormShare platform, which guarantees data security and real-time monitoring [39].

Enumerators were purposively recruited based on prior experience in market surveys and familiarity with local languages. Recruited enumerators underwent three days of training covering the study objectives, a detailed review of the data collection tool, standardized observation techniques, and ethical considerations. After training, the enumerators conducted a pretest to familiarize themselves with the tool. All food retail outlets within the specified administrative ward boundaries were assessed using the administrative boundary approach for food environment assessment [20]. The food retail mapping in each area was analyzed from 08:00h to 18:00h every day to ensure that all food retailers were mapped, regardless of their operating hours or mode. Food retailers known for operating either in the morning or late afternoon were specifically target within the time they operate.

For this study, a food retailer was defined as any individual or group of individuals selling food items, either exclusively or alongside non-food goods [38]. Retail typologies were derived from existing literature and included: *1*) butchers, *2*) cereal shops, *3*) cooked food street retailers, *4*) farmgate sales, *5*) home-based retailers, *6*) kiosks, *7*) mobile retailers, *8*) mom-and-pop shops, *9*) restaurants, *10*) stalls/tabletops, *11*) supermarkets, and *12*) wholesalers. A detailed description of the retail types is presented in Supplementary Material A and was adapted from [38]. All identified outlets were geocoded, with mobile retailers georeferenced at their location during encounters. Each outlet was categorized by typology. Observational data were collected on food groups and individual food items sold, retailer gender, and the shelf space occupied by food at the outlet to show whether the retailers sell only food (100%) or food together with other nonfood items.

### Data processing to compute different indices

#### Retail food environment quality indices

Data processing was conducted in R (version 4.4.2; R Foundation for Statistical Computing, Vienna, Austria) to compute 3 different retail food environment quality indices: the Healthy Retail Food Environment Score (HRFES), the Unhealthy Retail Food Environment Score (URFES) and the Retail Food Environment Quality Index (RFEQI). Concepts used for assessing dietary quality can be adapted to the retail food environment to inform measures of retail food environment quality, which is associated with individual diet quality [29]. The DQQ, originally developed as a tool for dietary assessment, has been proven to be a viable tool for food environment assessment, applicable in LMIC contexts [36].

In this paper, we adopt three indices validated for dietary intake assessment, derived from the DQQ—the GDR score, the NCD-risk score, and the NCD-protect score—to measure retail food environment quality [37,40]. These indices, validated as global dietary assessment tools, are based on binary food group counts [37]. Within the retail context, they are operationalized as the presence or absence of a particular food group at the retail level. The GDR indicators distinguish between food groups categorized as: NCD-protect (previously referred to as GDR-healthy): foods that protect against NCDs and should be promoted, and NCD risk (previously referred to as GDR-limit): foods that increase the risk of NCDs and should be limited [41].

The GDR indicators are based on global recommendations on healthy diets and evidence on the relationship between diets and NCDs [[42], [43], [44], [45], [46], [47], [48]]. The computation of these indices in the context of retail food environment quality is described as follows:

##### HRFES

The HRFES has been adapted from NCD-protect, which was developed based on five global guidelines on nutritious foods for healthy diets, which are important in protecting against diet-related NCDs [37,41]. The HRFES measures the extent to which the retail food environment offers healthy food variety. It is based on the following five food categories: fruit, legumes, nuts and seeds, vegetables, and whole grains. The foods were grouped into nine food groups (Table 1 [37,41]). These food groups were scored on a dichotomous scale, with a value of one indicating the presence and zero indicating the absence of the food category at the retail level. The maximum score possible was nine, if a retailer had all nine food groups listed, and the minimum score is zero if a retailer does not sell any of the listed food groups. The HRFES can also be used to measure the availability of nutrient-dense foods in a particular retail food environment, contributing to efforts to enhance healthy, sustainable food systems.

**TABLE 1:** Food groups for computing retail food environment quality indices

Retail food environment Quality indices	Food groups	Scoring
Healthy Retail Food Environment Score (HRFES)		0–9
	Dark-green leafy vegetables	0/1
	Vitamin A–rich, orange-colored vegetables	0/1
	Other vegetables	0/1
	Vitamin A–rich fruits	0/1
	Citrus fruits	0/1
	Other fruits (including red/purple/blue fruits)	0/1
	Legumes	0/1
	Nuts/seeds	0/1
	Whole grain	0/1
Unhealthy Retail Food Environment Score (URFES)		0–9
	Sodas/sugar-sweetened beverages	0/1
	Baked/grain-based sweets	0/1
	Other sweets	0/1
	Processed meats (double weight)	0/2
	Unprocessed red meat	0/1
	Deep-fried foods	0/1
	Food from a fast-food restaurant, or Instant noodles	0/1
	Packaged salty snacks	0/1
Retail Food Environment Quality Index (RFEQI)	HRFES − URFES + 9	0–18

The table has been adapted from global dietary recommendation score indices for diet quality assessment [37,41].

##### URFES

The URFES has been adapted from NCD risk, which was developed based on six global dietary guidelines on foods to limit due to their contribution to NCDs [37,41]. The availability of such foods within a particular retail food environment may encourage their consumption, thereby increasing diet-related NCD risks [49]. The URFES covers processed meats, red meat, and other food groups that are high in salt, sugar, and fat. For scoring, the foods are grouped into eight food groups as shown in Table 1. The study scored these food groups on a dichotomous scale, with a value of one indicating their presence and zero indicating their absence at the retailer, except for the processed meats category, which was scored as two for presence and zero for absence due to its high association with NCDs [45]. The NCD-risk score also serves as an indicator of ultraprocessed foods [41]. Hence, URFES could also be used as a proxy for characterizing the obesogenic food environment. The score ranges from zero for retailers that do not sell any food group in this category to nine for retailers that sell all eight food groups. Higher scores indicate a greater presence of unhealthy food groups, which contribute to a higher NCD risk.

##### RFEQI

The RFEQI is derived from the GDR score, which was initially created to assess compliance with global dietary guidelines [37,41]. The RFEQI helps to assess the extent to which the retail food environment offers a variety of healthy foods or lacks them. The RFEQI was computed by subtracting the number of food groups under URFES from the number of food groups under HRFES, then adding 9 to avoid using negative values in the index [41]. The RFEQI range is 0−18.

#### Retail neighborhood buffer distance

Retail neighborhood buffers of 50 m, 100 m, and 200 m were generated around each retail food outlet to characterize its surrounding food environment. For each buffer, all retail outlets located within the specified distance, including the focal outlet, were identified. The presence of unique healthy and unhealthy food groups across these outlets was then determined, and the HRFES, URFES, and RFEQI were recalculated at the buffer level using the scoring approach described earlier. This allowed us to assess the quality of the retail food environment surrounding each outlet rather than relying solely on the characteristics of individual retailers. A flexible function in Python was developed using GeoPandas v0.14.3 to aggregate different indices according to their spatial proximity, while specifying the buffer distance and the data frame [20].

### Data analysis

Data analysis was performed in R (Version 4.4.2; R Foundation for Statistical Computing), except for the spatial analysis of retail outlet dispersion and concentrations of healthy and unhealthy food groups (hot spots and cold spots), which were conducted in Python version 3.13.1 (Python Software Foundation). The analysis of food environment quality indices was stratified by location (rural compared with urban). The R packages used for cleaning and data analysis included dplyr, readxl, stringr, Hmisc, gtsummary, MASS, lmtest, car, pscl, patchwork, ggtext, corrplot, and ggplot2. Descriptive statistics included means, SDs, and frequencies. Fisher’s exact test and Kruskal-Wallis rank sum test were employed to examine differences in retail food environment quality indices between rural and urban retail food environments.

To assess the internal consistency, reliability, robustness, and stability of the indices, a sensitivity analysis was conducted using a Monte Carlo simulation. We performed 100,000 simulations, randomly selecting 70% of the dataset for each analysis. The results were visualized using a histogram, where a normal distribution indicates the reliability and robustness of the developed indices.

Fixed-effect regression analysis was conducted to examine the association between retail food environment quality indices: RHFES, RUFES, and RFEQI and retail food environment characteristics, including retail typology, retailer gender, and retail food space. This was based on the equation below:

$$Y_{i}=\sum_{j=1}^{11}\beta_{1j}\;{RTYPE}_{ij}+\sum_{g=1}^{4}\beta_{2g}\;{RGENDER}_{ig}+\sum_{l=1}^{4}\beta_{3l}\;{RSPACE}_{il}+\beta_{4}{RLOC}_{i}+\epsilon_{i}$$

Where *Y*_*it*_ denotes the retail food environment quality outcome (RHFES, RUFES, and RFEQI), R*TYPE*_*ij*_ represents the 12 retail typologie*s, RGENDER*_*ig*_ represents the retailer’s gender_*,*_ *RSPACE*_*il*_ represents the retail outlet food space*, RLOC*_*i*_ denotes the location of the retail outlet (rural/urban)*,* and *ϵ*_*i*_ represents the error term.

Spatial analysis was undertaken to evaluate the degree of clustering or dispersion of retail outlets and associated food group categories (healthy or unhealthy). Multi-distance spatial clustering was assessed using Ripley’s K function to determine whether retail outlets in rural and urban areas exhibit significant clustering, dispersion, or spatial randomness. This analysis was implemented in Python using the distance statistics K-test function. The horizontal axis shows distance in meters, whereas the vertical axis displays K(r), which represents the expected number of neighboring retail outlets within a radius r compared with complete spatial randomness (CSR). The orange curve corresponds to the empirical K function estimated using isotropic edge correction. The two black curves mark the upper and lower limits of the 99% Monte Carlo simulation envelope generated from 499 edge corrected simulations, and the shaded band indicates the range of values anticipated under spatial randomness. When the empirical K(r) exceeds the upper envelope, the pattern reflects statistically significant clustering at that distance, whereas values falling below the lower envelope indicate significant spatial regularity or dispersion at the 1% pointwise level. Z max identifies the greatest deviation from spatial randomness across all evaluated distances, and r peak indicates the distance at which this maximum deviation occurs [50].

Clustering of healthy or unhealthy food categories to identify hot spots or cold spots for the respective groups was carried out in two stages. First, Global Morans’ I (spatial autocorrelation) analysis was performed to determine whether the food group categories are clustered, dispersed, or randomly distributed. This involved estimating the Moran’s I coefficient value along with its *P* value. The Global Morans’ I coefficient ranges from ‒1 to 1, with a value of 0 indicating CSR. A positive value with a *P* value < 0.05 suggests significant spatial clustering of similar values (high scores tend to be near other high scores; low scores near other low scores), with values close to 1 indicating strong clustering. Conversely, negative values with a *P* value < 0.05 indicate significant spatial dispersion (high scores tend to be near low scores and repel other high scores), with values nearer to ‒1 indicating strong dispersion [[51], [52], [53]]. The analysis was conducted using the pysal package in Python.

Second, after ruling out CSR using the Global Moran’s statistic and to identify hot spots with more clarity, we employed local indicators of spatial autocorrelation (LISA) to identify locations within the respective retail food environments that show significant clusters of high-high and low-low diversity in the assessed indices, relative to neighboring retailers. It also highlights locations with high-low and low-high diversity across different retail food environment zones, where values differ significantly from those of surrounding areas. Zones that are not significant, indicating random distribution of retailers, were also determined based on values in nearby areas within the food environments.

## Results

### Descriptive characteristics of food retailers

Approximately 57% of the retail outlets exclusively sold food, whereas the remaining ones combined food with nonfood items. Across both regions, the majority of retailers were female (71%). The predominant retail typologies in both rural and urban retail food environments were cooked food street retailers, kiosks, and stalls (Table 2).

**TABLE 2:** Characteristics of food retailers in the urban and rural food environment

Characteristic	Overall *n* = 2086	Urban *n* = 1192	Rural *n* = 894	*P* value^1^
**Food retail space**^2^				0.001
100% food space	57.1	58.2	55.7	
Between 75% and 100%	27.0	26.8	27.4	
Between 75% and 50%	10.9	8.9	13.7	
Between 50% and 25%	3.5	4.2	2.4	
Below 25%	1.4	1.9	0.8	
**Gender of retailers**^3^				0.022
Female	71.3	71.8	70.7	
Male	25.4	24.0	27.2	
Mixed gender	2.1	2.5	1.6	
Multiple females	0.8	1.3	0.2	
Multiple males	0.4	0.4	0.3	
**Retail typology**				<0.001
Cooked food street retailers	25.4	31.7	16.9	
Kiosk	31.9	26.8	38.8	
Stall/tabletop	26.5	25.1	28.3	
Mobile retailers	6.6	9.7	2.5	
Butcher	3.9	3.3	4.7	
Cereal shop	1.7	1.2	2.5	
Wholesalers	1.4	1.2	1.8	
Modern restaurant	0.9	0.3	1.7	
Home-based retailers	0.6	0.3	0.9	
Mom-and-pop shops	0.2	0.2	0.2	
Farmgate sale	0.6	0.1	1.3	
Supermarket	0.2	0.1	0.4	

1 Fisher’s exact test for comparing proportions in urban and rural. P-Values of 0.05 and below were considered significant.

2 Retail food space is defined as the share of the retail outlet occupied by food.

3 Gender of the person selling food at the retail outlet, mixed gender indicate the retail outlet had both genders; multiple females indicate the retail outlet has >1 female, and multiple males indicate the retail outlet had >1 male. Retail typology description presented in Supplemental Material A.

### Retail food environment quality

**FIGURE 2:**
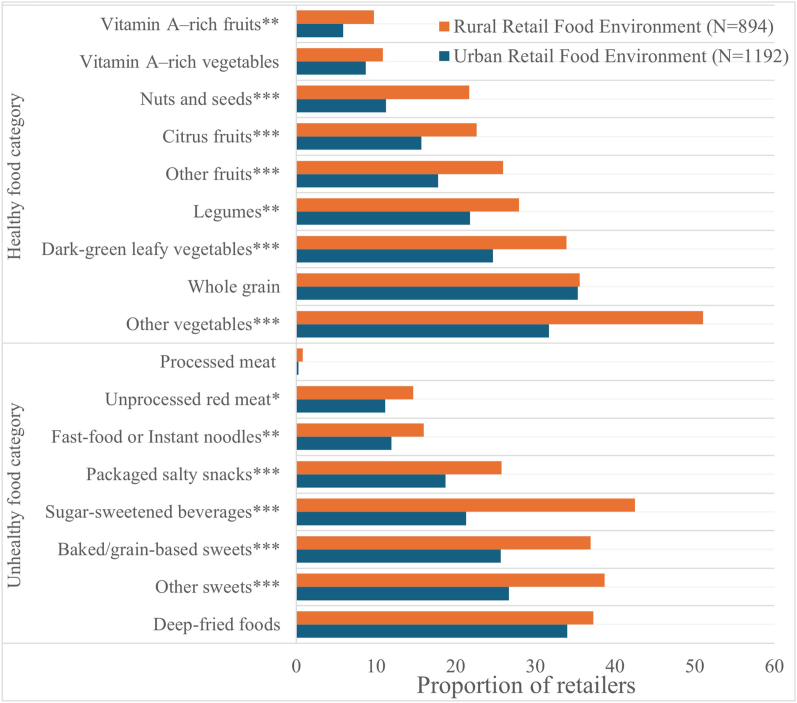
Healthy and unhealthy food groups offered by retailers in the urban and rural retail food environment. Fisher’s exact test was used to compare the proportion of retailers in the urban and rural areas selling different food groups. ∗∗∗indicates *P* value < 0.001, ∗∗indicates *P* value < 0.01, and ∗indicates *P* value < 0.05.

There were differences in the proportion of retailers selling different food groups in the rural and urban retail food environment (Figure 2). Generally, rural retailers offered a high proportion of both healthy and unhealthy food groups. For healthy food groups, the most available foods were whole grains, other vegetables, and dark-green leafy vegetables. Vitamin A–rich foods were the least available in both areas, as indicated by the low number of retailers selling vitamin A–rich, orange-colored vegetables and fruits.

For unhealthy food groups, the proportion of rural retailers selling soda/sugar-sweetened beverages was twice that of urban retailers. Other unhealthy food groups that recorded a high proportion of retailers included deep-fried foods, other sweets, and baked or grain-based sweets. Processed meat had the least proportion of retailers in both urban and rural food environments.

**FIGURE 3:**
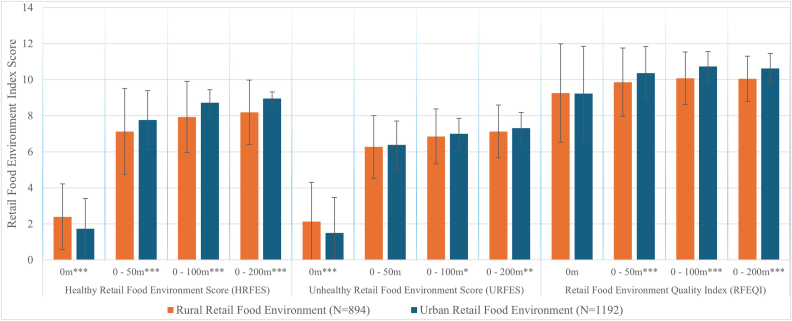
Indices for characterizing healthy or unhealthy food diversity across rural and urban retail food environments. The error bars indicate standard deviation. Welch 2-sample t-tests were used to compare the mean retail food environment index score in the rural and urban food environments at different retail neighborhood distances. Max score for Healthy Food Environment Score is 9, higher scores indicate more healthy foods; Unhealthy Food Environment Score max score 9, higher scores indicate more unhealthy foods; Retail Food Environment Score max score 18, higher scores indicate better overall food environment quality. ∗∗∗indicates *P* value < 0.001, ∗∗indicates *P* value < 0.01, and ∗indicates *P* value < 0.05.

The results of the different indices in the rural and urban retail food environment, both at the retail and at different retail neighborhoods’ level, are presented in Figure 3. Both healthy and unhealthy food groups were present in rural and urban retail food environments. The mean HRFES was slightly higher in rural areas (2.01 ± 1.77) than in urban areas (1.77 ± 2.07). Rural retailers recorded significantly higher mean scores for both HRFES and URFES compared with their urban counterparts (*P* < 0.001).

However, when considering retail neighborhood radii of 50 m, 100 m, and 200 m, urban HRFES and URFES values surpassed those of rural areas. Expanding the neighborhood radii further accentuated the diversity of the urban retail food environment across all 3 indices. The RFEQI was generally low across both rural and urban food environments. The mean RFEQI score at the retailer level was similar in rural and urban retail food environments. By increasing the retail neighborhood radii, the RFEQI increased significantly (*P* < 0.001) in the urban environment compared with the rural one.

**FIGURE 4:**
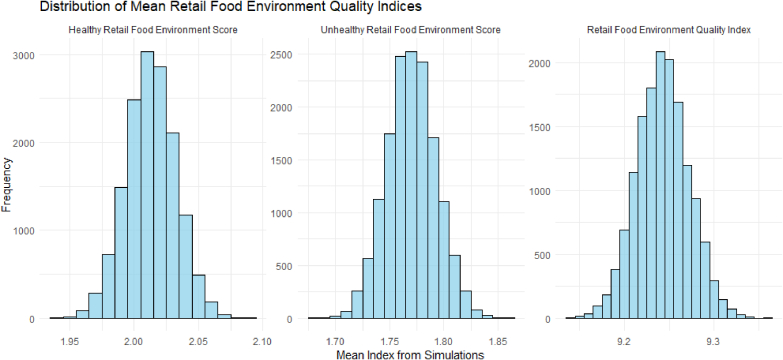
Monte Carlo simulation to test the sensitivity and stability of the indices for measuring the quality of the food environment.

Monte Carlo simulation showed a normal distribution curve for the various indices (Figure 4). This indicates that the developed indices are sufficiently reliable and robust to evaluate the quality of the retail food environment in terms of the availability of healthy or unhealthy food groups.

### Regression analysis on the association of food environment quality indices and the retail characteristics

Table 3 presents the results of the fixed-effects regression analysis examining associations between retail characteristics and the food environment quality indices. Location was significantly associated (*P* < 0.01) with all retail food environment quality indices. Using the rural retail food environment as the reference category, the urban retail food environment showed weaker associations with both HRFES and URFES. Specifically, urban retailers had significantly lower RFEQI scores compared with rural retailers’ incidence rate ratio (IRR) = 0.96, *P* < 0.01. However, when retailer neighborhoods of 50 m and beyond were considered, the associations of urban food environment with HRFES and RFEQI exceeded those observed in rural areas (Supplementary Material B).

**TABLE 3:** Fixed-effects regression on the relationship between retail food environment indices and retail food environment characteristics

Characteristic	HRFES (*n* = 2086)			URFES (*n* = 2086)			RFEQI (*n* = 2086)		
	IRR	95% CI	*P* value	IRR	95% CI	*P* value	IRR	95% CI	*P* value
**Retail location**									
Rural	Reference	—		—	—		—	—	
Urban	0.75	0.71, 0.80	<0.001	0.90	0.84, 0.96	0.002	0.96	0.94, 0.99	0.019
**Retail food shelf space**									
100% food space	Reference	—		—	—		—	—	
Between 75% to 99%	1.24	1.13, 1.35	<0.001	2.00	1.80, 2.23	<0.001	0.94	0.90, 0.97	0.002
Between 50% to 74%	1.13	1.00, 1.27	0.042	1.85	1.63, 2.09	<0.001	0.94	0.88, 0.99	0.025
Between 25 to 49%	0.65	0.51, 0.81	<0.001	1.40	1.16, 1.69	<0.001	0.93	0.86, 1.02	0.130
Below 25%	0.31	0.18, 0.49	<0.001	1.04	0.75, 1.43	0.800	0.92	0.81, 1.05	0.200
**Retailer’s gender**									
Female	Reference	—		—	—		—	—	
Male	0.83	0.77, 0.90	<0.001	1.10	1.02, 1.19	0.009	0.94	0.90, 0.97	<0.001
Mixed gender	1.37	1.12, 1.65	0.001	1.35	1.12, 1.62	0.002	0.99	0.89, 1.09	0.800
Multiple females	1.04	0.72, 1.45	0.800	10	0.64, 1.56	>0.900	0.99	0.85, 1.16	>0.900
Multiple males	0.68	0.31, 1.27	0.300	0.81	0.42, 1.56	0.500	0.98	0.77, 1.22	0.800
**Retail typology**									
Mobile retail	Reference	—		—	—		—	—	
Butcher	0.10	0.04, 0.20	<0.001	2.90	1.95, 4.30	<0.001	0.87	0.79, 0.96	0.005
Cereal shop	2.51	1.89, 3.33	<0.001	3.09	1.98, 4.80	<0.001	1.09	0.97, 1.22	0.150
Cooked food street vendor	2.04	1.68, 2.49	<0.001	3.53	2.54, 4.91	<0.001	1.02	0.96, 1.08	0.600
Farmgate sale	2.20	1.43, 3.28	<0.001	0.00	0.00, 0.00	>0.900	1.16	0.98, 1.38	0.084
Home vendor	1.65	0.99, 2.59	0.042	7.75	4.89, 12.3	<0.001	0.81	0.65, 0.99	0.046
Kiosk	1.80	1.47, 2.22	<0.001	8.81	6.33, 12.2	<0.001	0.74	0.69, 0.79	<0.001
Supermarket	2.32	1.24, 3.99	0.005	11.30	6.96, 18.5	<0.001	0.62	0.41, 0.88	0.012
Mom-and-pop shops	2.56	1.33, 4.48	0.002	8.18	4.53, 14.8	<0.001	0.86	0.59, 1.20	0.400
Modern restaurant	2.86	2.06, 3.94	<0.001	11.30	7.47, 16.9	<0.001	0.89	0.75, 1.04	0.200
Stalls/tabletop	3.15	2.61, 3.85	<0.001	0.99	0.69, 1.41	>0.900	1.19	1.12, 1.27	<0.001
Wholesalers	1.20	0.80, 1.74	0.400	3.50	2.21, 5.55	<0.001	0.94	0.82, 1.07	0.400
**Model fit**									
PseudoR^2^	0.109			0.373			0.086		
AIC	7168			5593.4			9259		

Retail food shelf space = the proportion of the outlet space occupied by food. Retailer’s gender = gender of the person selling food at the retail outlet, mixed gender indicate the retail outlet had both male and female persons at the outlet; multiple females indicate the outlet has >1 female, and multiple males indicate the outlet had >1 male.

Abbreviations: AIC, Akaike Information Criterion; CI, confidence interval; RFEQI, Retail Food Environment Quality Index; HRFES, Healthy Retail Food Environment Score; IRR, incidence rate ratio; URFES, Unhealthy Retail Food Environment Score. P-Values of 0.05 and below were considered significant

Food retail shelf space was positively associated with HRFES and URFES, with larger retail spaces contributing significantly to higher RFEQI. However, when retail neighborhoods were considered, retail space was not significantly associated with any of the food environment quality indices (Supplementary Material B).

Retailer’s gender was significantly associated with all 3 indices. Compared with female retailers, male retailers were less likely to offer healthy foods (HRFES) (IRR = 0.83, *P* < 0.001) and RFEQI (IRR = 0.94, *P* < 0.001), but more likely to offer unhealthy foods (URFES) (IRR = 1.1, *P* <0.05). Retail outlets operated by mixed-gender retailers were significantly more likely to offer both healthy foods. These associations were not significant when retail neighborhood distances of 100 m radius and beyond were considered (Supplementary Material B).

Retail typology showed the strongest associations with all indices. Compared with mobile retailers, stall/tabletop retailers were significantly more likely to achieve higher HRFES (IRR = 3.15, *P* < 0.001) and RFEQI (IRR = 1.19, *P* < 0.001). Other retail categories positively associated with HRFES and RFEQI included cereal shops, cooked food street retailers, farmgate sellers, mom-and-pop stores, and modern restaurants. By contrast, butcher shops were significantly associated with lower HRFES (IRR = 0.10, *P* < 0.001) and RFEQI (IRR = 0.87, *P* < 0.05). Strong positive associations with URFES were observed for supermarkets (IRR = 11.30, *P* < 0.001), kiosks (IRR = 8.81, *P* < 0.001), mom-and-pop shops (IRR = 8.18, *P* < 0.001), and modern restaurants (IRR = 11.30, *P* < 0.001), all of which were concurrently associated with significantly lower RFEQI. At retail neighborhood distances of 100 m radius and beyond, only home retailers and farmgate sellers remained significantly associated with RFEQI (Supplementary Material B).

### Spatial analysis of the retail food environment

#### Multidistance spatial cluster analysis

**FIGURE 5:**
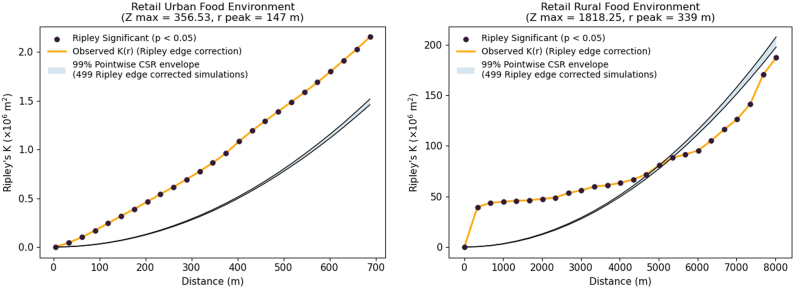
Measure of retail spatial clustering/dispersion in the rural and urban retail food environment using Ripley’s K function. The empirical K(r) (orange line) is plotted against distance (meters) and compared with the 99% Monte Carlo simulation envelope (black lines and shaded area) under the assumption of complete spatial randomness (CSR). Values above the upper envelope indicate significant clustering, values below the lower envelope indicate dispersion, with Z max representing the maximum deviation and r peak the distance at which it occurs.

The spatial distribution of retail outlets in both urban and rural food environments exhibited clustering, as indicated by observed K values exceeding expected K values (Figure 5). In the urban food environment, the observed K function remained above the CSR envelope across the entire evaluated range up to ∼700 m, indicating statistically significant clustering of retail outlets at all examined distances. The maximum deviation from CSR occurred at 147 m (Z max = 356.53), identifying this as the dominant clustering scale. The shorter maximum distance reflects the smaller administrative extent of the urban food environment relative to the rural food environment rather than a methodological constraint. Overall, the results indicate a compact and consistently aggregated retail outlet distribution, with clustering strongest at neighborhood-level distances. In a rural food environment, the observed K function also exceeded the CSR envelope at short distances, with a sharp increase between 0 m and 1000 m. Significant clustering persisted up to ∼4000 m. The strongest departure from spatial randomness occurred at 339 m (Z max = 1818.25), indicating a broader dominant clustering scale and substantially greater clustering intensity than in the urban food environment. Between roughly 4500 m and 5500 m, the observed curve approached the CSR envelope, suggesting spatial randomness at these broader scales. Beyond ∼5500 m, the observed K fell below the envelope, indicating significant spatial dispersion. Overall, both areas exhibited clear local clustering of retail outlets. However, the rural food environment demonstrated stronger and more spatially extensive clustering followed by large-scale regularity, whereas the urban food environment showed persistent small-scale clustering without evidence of large-scale dispersion within its administrative boundary.

#### Global Moran’s I analysis

The spatial autocorrelation showed that spatial clustering was more evident in the rural retail food environment, where all indices exhibited statistically significant clustering, although the magnitude of clustering, as indicated by the Moran’s I coefficients, was generally weak (Table 4). In the urban retail food environment, only URFES displayed significant spatial clustering. Across both rural and urban contexts, URFES demonstrated the highest Moran’s I coefficient, indicating the strongest tendency toward spatial clustering. This pattern suggests that while clustering exists, the relatively weak magnitude reflects considerable heterogeneity in the spatial distribution of retail outlets across both settings.

**TABLE 4:** Moran spatial autocorrelation (Global Moran’s I) results for the different retail food environment indices across the rural and urban food environment

Food environment indices	Rural retail food environment		Urban retail food environment	
	Morans I	*P* value	Morans I	*P* value
Healthy retail food environment score	0.099	0.001	0.000	0.480
Unhealthy Retail Food Environment Score	0.125	0.001	0.073	0.002
Retail Food Environment Quality Index	0.105	0.001	0.024	0.128

The Global Moran’s I coefficient ranges from ‒1 to 1, with a value of 0 indicating complete spatial randomness. Positive values with a *P* value < 0.05 indicate spatial clustering, with values closer to 1 reflecting stronger clustering. Negative values with a *P* value < 0.05 indicate significant spatial dispersion, with values closer to ‒1 indicating stronger dispersion.

#### LISA

**FIGURE 6:**
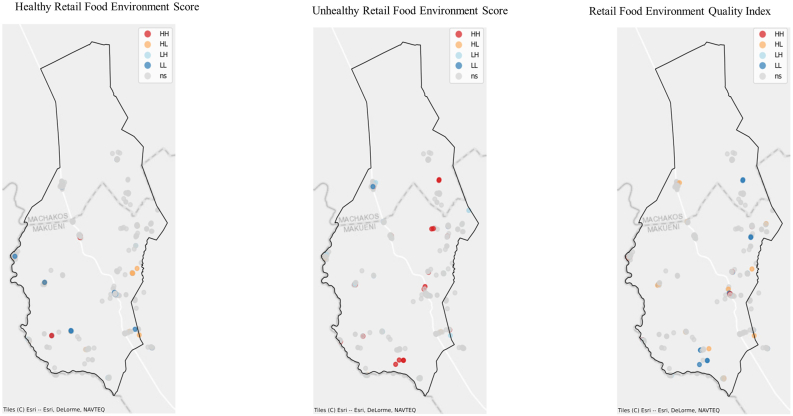

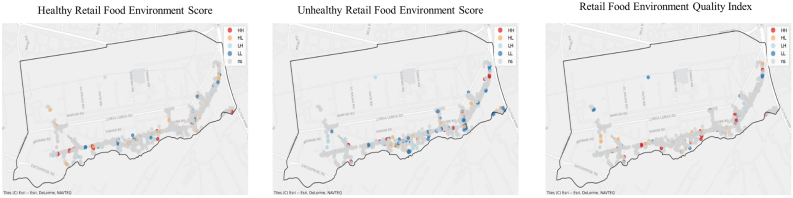
(A) Local indicators spatial autocorrelation results for the rural retail food environment (Kiima kiu ward in Makueni county, Kenya, covering 446.2 km^2^), indicating hot and cold spots for the different indicators. HH: (higher values clustering together); HL: (higher values clustering around low values); LH: (low values clustering around high values); LL: (low values clustering around low values). (B) Local indicators spatial autocorrelation results for the urban retail food environment. (Viwandani ward in Nairobi county, Kenya, covering 5.0 km^2^), indicating hot and cold spots for the different indicators. HH: (higher values clustering together); HL: (higher values clustering around low values); LH: (Low values clustering around high values); LL: (Low values clustering around low values). HH, high-high; HL, high-low; LH, low-high; LL, low-low; ns, not significant.

Figure 6 A and B present the results of the LISA analysis for both rural and urban retail food environments. Overall, clustering of the different indices was largely insignificant in both settings. Nonetheless, the urban food environment exhibited a greater number of high-high clusters compared with the rural food environment. Across both contexts, unhealthy food groups displayed more high-high clusters than healthy food groups. Notably, nonsignificant areas dominated much of the spatial distribution maps, indicating the absence of statistically significant spatial autocorrelation in most locations. This pattern suggests that the majority of retailers offering different food groups are more randomly distributed rather than forming distinct spatial clusters.

## Discussion

The study provides a novel framework for monitoring the quality of the retail food environment, which serves as the primary food source for households in both rural and urban areas [8,54]. The rural retailers in this study displayed greater diversity across both healthy and unhealthy food groups compared with their urban counterparts; however, when the retail neighborhood was considered, urban areas showed higher diversity. Retail typologies were also found to be strongly associated with retail food environment quality, underscoring the potential for targeting specific typologies to promote healthier food availability. In addition, spatial analyses identified locations with high or low concentrations of healthy and unhealthy food groups. These insights highlight geographic disparities in accessing healthy and unhealthy foods and provide opportunities for context-specific policy interventions tailored to the unique dynamics of rural and urban food environments.

### Retail food environment quality

Individual retailers in rural areas offered a wider variety of healthy food groups than their urban counterparts. However, when the retail neighborhood within a 50–200 m radius was considered, urban areas demonstrated significantly greater diversity of healthy food groups (HRFES). This result aligns with findings from Ethiopia, Kenya, and Vietnam, where, according to a Nutrition-Sensitive Food Environment Index, urban retail neighborhoods exhibited higher diversity than rural areas, despite rural retailers individually offering more variety [20]. Another study in Bangladesh also reported that increasing retail distance increases the density of both markets and groceries, which in turn influences retail food environment quality [55]. The findings underscore the importance of evaluating the food environment beyond the individual retail outlet, as neighborhood-level assessments provide a more comprehensive understanding of healthy food diversity.

Between the healthy food groups, (pro-)vitamin A–rich fruits and vegetables were the least available. Despite vitamin A deficiency remaining a major public health concern in SSA, with a prevalence of ∼48% [56], limited availability of these foods has been reported in several countries [57]. This is particularly concerning given evidence that greater vegetable availability is associated with higher purchase and consumption likelihood [18,58]. These findings highlight the urgent need for interventions to increase the availability of vitamin A–rich foods and other nutrient-dense produce to improve diet quality and mitigate micronutrient deficiencies.

The “URFES” revealed similar urban–rural patterns, although differences were smaller than those observed in the HRFES. This convergence suggests a growing similarity in exposure to unhealthy foods across rural and urban contexts, potentially heightening diet-related NCD risks in both areas. Deep-fried foods and baked or grain-based sweets were especially prevalent, both commonly prepared with artificial *trans* fats and inexpensive shelf-stable oils linked to increased cardiovascular disease risk [44,59,60]. Sugar-sweetened beverages were also widely available, particularly in rural areas, consistent with evidence of rising sugar-sweetened beverages availability and consumption in LMICs [61]. Sugar-sweetened beverage consumption is strongly associated with obesity, heart disease, and type 2 diabetes [44,62]. The increased penetration of unhealthy food groups into both rural and urban retail systems may accelerate the nutrition transition and contribute to the rising NCD burden in SSA.

These findings underscore the urgency of policy measures to limit the availability of unhealthy foods while promoting access to healthy alternatives. Fiscal measures, such as taxation of unhealthy foods and sugar-sweetened beverages, have proven effective in discouraging consumption [63,64]. An analysis of sugar-sweetened beverages imports in 44 LMICS reported that every 1% rise in tariffs was associated with a 2.9% drop in imports of sugar-sweetened beverages [65]. Complementary strategies include restrictions on unhealthy food marketing [66], subsidies to reduce the price of healthy foods [63], and front-of-pack warning labels to guide consumer choice [33]. However, the effectiveness of such labeling policies may be limited among populations with low literacy levels, emphasizing the need for broader food system interventions that do not rely solely on individual knowledge or decision-making capacity. Effective interventions to improve retail food environment quality must therefore simultaneously promote healthy foods and limit unhealthy options, with particular attention to low socioeconomic groups who face disproportionate exposure to unhealthy retail environments [66,67]. In addition, the school food environment also needs strict interventions to keep away unhealthy foods since consumption decisions of adolescents who mainly attend school are mostly driven by accessibility of foods around the school environment [68].

The rapid rise in BMI (in kg/m^2^) values among rural populations in SSA [7] further highlights the need for close monitoring of the rural food environment, where these consumers predominantly source their diets. Preventing the shift from undernutrition to overconsumption of calorie-dense, nutrient-poor foods is critical to mitigating the double burden of malnutrition. Ongoing initiatives—such as the Access to Nutrition Initiative, which independently evaluates packaged foods and beverages in East African markets—reveal that only a minority of products meet healthiness thresholds, with just 33% of Kenyan products achieving the Health Star Rating threshold of 3.5 of 5 [33,34]. Although nutrient profiling offers the most rigorous method of assessing food healthiness, it is resource-intensive and requires technical expertise. By contrast, the HRFES, URFES, and RFEQI developed in this study provide simple, quantitative tools for monitoring retail food environment quality. These indices are thus practical for routine surveillance and policy application in LMIC contexts.

### Contribution of retail characteristics to food environment quality

The results of this study showed that the rural retail level was positively associated with retail food environment indices compared with the urban retail level. However, when considering neighborhood-level effects, the urban retail food environment demonstrated significantly stronger associations with the indices. This pattern may be explained by the high concentration of retailers in urban settings, many of which offer a limited food diversity but differ in the products they sell. Collectively, this produces a higher overall diversity of both healthy and unhealthy food groups in urban areas [38]. Although neighborhood availability reflects the external food environment potential, personal food environment factors may shape people’s ability to access and utilize available foods. Socioeconomic status, purchasing power, mobility constraints, time availability, food preferences, and nutrition knowledge can influence how individuals navigate their food environments and translate availability into actual food acquisition [5]. Consequently, the observed neighborhood-level associations should be understood within a broader socioecological context in which retail exposure interacts with personal factors to influence access to healthy and unhealthy foods.

Gender showed a significant association with retail food environment quality: female retailers were more strongly associated with healthy food offerings, whereas male retailers were more commonly linked with unhealthy food options. This gendered differentiation reflects the retail typologies in which males and females are engaged. Males were more frequently associated with kiosks, butchers, and supermarkets—retail typologies that predominantly stock processed foods—whereas females were more often linked with stalls or tabletops, which largely sold fresh fruit and vegetables. Females’ role in the retail food environment is particularly important, as their services not only increase the availability of healthier foods but also support food convenience, which strongly influences household purchasing decisions [69]. These dynamics may account for the positive association between female retailers and higher HRFES scores. Recognizing this contribution is critical for policymakers seeking to design interventions that leverage females’ role in promoting healthier diets.

Associations between retail typologies and food environment quality were most evident at the individual retail level and diminished when considering the retail- neighborhood food environment. This finding underscores the importance of targeting interventions to specific retail typologies within each environment. Stalls demonstrated the strongest positive associations with HRFES and RFEQI, and a negative association with URFES, consistent with their focus on fresh produce. Interventions aimed at improving retail food environments should therefore prioritize increasing the prevalence of these typologies. Conversely, supermarkets exhibited the strongest associations with unhealthy food availability, followed by kiosks, whereas contributing the least to RFEQI. Despite supermarkets’ gradual inclusion of fresh produce in urban food environments, those in rural and informal urban settings continue to predominantly sell processed and ultraprocessed foods that are high in sugar, salt, and saturated fats. This aligns with findings from Kenya, where supermarket purchases have been linked to overweight, obesity, and diet-related NCDs [70,71]. Similarly, evidence from Ghana and South Africa has demonstrated supermarkets’ central role in facilitating access to obesogenic foods [31]. Although household reliance on supermarkets in SSA remains relatively low, their expansion into both rural and urban markets is accelerating [21,27,72,73]. Studies have also reported that supermarkets are useful for increasing the food diversity available to consumers and increasingly stock fresh produce, though at higher prices than traditional markets [71,74].

These findings underscore the need for deliberate efforts to increase the availability of fresh produce in supermarkets, especially in rural areas, as a strategy to enhance retail food environment quality and mitigate the growing NCD burden. For such an approach to succeed, it requires policymakers to adopt a bottom-up approach, where community members are engaged to drive the changes needed to improve their food environments [75]. Our findings differ from those in high-income countries, which report that supermarkets are an important source of healthy foods such as fruits, vegetables, and other nutritious options. Access to these outlets has been associated with higher consumption of these foods in some populations [76,77]. In LMICs, few studies have associated supermarkets with the dietary diversity of consumers [74]. With this finding, it would be important, before classifying retail typology as healthy, to consider the food diversity sold, as this differs markedly between different regions.

### Spatial distribution of food environment indices

Spatial autocorrelation offers critical insights for designing retail food environment interventions by revealing the spatial organization, clustering, and dispersion of retailers and the distribution of healthy and unhealthy food groups. Beyond generating global measures, it provides localized evidence on how retail food environment indicators and indices perform across different settings. In this study, clustering of retailers was observed in both urban and rural retail food environments, suggesting that retailers tend to concentrate in specific locations. Similar findings have been reported in Ghana and South Africa, where retailers predominantly operate along main access roads [31], and in Malawi, where clusters of retailers were documented in both rural and urban settings [29]. Factors influencing such clustering include accessibility for customers, availability of space, proximity to major road networks, and economies of scale benefits [78]. Understanding these drivers is critical for developing strategies to attract retailers to underserved areas, thereby addressing food deserts and promoting equitable access to healthy foods.

Spatial autocorrelation analysis also makes it possible to identify hot spots and cold spots of retail food environment indices, thereby highlighting areas of relative abundance or scarcity of specific food groups. Such information is valuable for policymakers when determining where to promote healthier options or where to intervene to reduce the prevalence of unhealthy food outlets. Other studies on spatial food group distribution have focused solely on the distribution within the food environment, without considering areas where there is a concentration of healthy or unhealthy foods [3,21,29]. Another study only reported distances from households associated with hot or cold spots for the food groups of interest [18], without identifying specific locations corresponding to hot spots or cold spots of food groups. Recognizing spatial concentrations of unhealthy food groups, for instance, provides opportunities to target these locations with interventions that expand healthier alternatives. Conversely, identifying cold spots for healthy foods can guide strategies to improve availability and accessibility in underserved areas.

Importantly, the spatial findings complement the regression results by showing that the quality of retail food environments is not only shaped by retailer and typology characteristics but also by where these retailers are located and how they cluster across space. Together, these dimensions emphasize that interventions to improve food environment quality must integrate both retailer level and spatial considerations to be effective and context-specific.

### Limitations of the study

This study did not directly examine the association between the retail food environment and consumers’ diet quality. Nonetheless, the indices developed have demonstrated reliability and can be applied across diverse contexts. Importantly, they were adapted from a validated index for dietary quality assessment, ensuring conceptual robustness. Given the well-established evidence that food environments substantially influence diet quality [29,79,80], these indices provide a credible proxy for monitoring retail food environments and offer an evidence base for inferring potential dietary impacts in the assessed areas.

Another limitation concerns the exclusion of certain food groups—such as eggs, dairy, fish, and poultry—that were present in the study environments. However, the food groups included in the indices were specifically selected for their protective or harmful roles in relation to NCD risk [37,41]. Although the omitted groups remain important for overall dietary adequacy, they have not been consistently linked to NCD risk [42,43,45,47,48]. The indices, therefore, remain highly relevant for policy interventions aimed at addressing NCD prevention and control, which was the central focus of this study. However, since the index is limited to the NCD burden, other indices that can assess multiple malnutrition burdens need to be developed.

Finally, although the indices represent a novel and practical approach to quantifying food environment quality, further research is recommended to validate the tool in diverse settings. Validation studies should link these indices to observed dietary intake, nutritional status, and health outcomes. Additionally, the validation could include setting the threshold for RFEQI. This would strengthen their utility for surveillance and policy applications. Additionally, subsequent studies should integrate spatially explicit retail food environment measures with consumer dietary intake and NCD outcome data. This linkage would enhance understanding of how spatial disparities in healthy and unhealthy food availability influence diet quality and health risks, thereby improving the design of targeted interventions.

In conclusion, this study makes a unique and novel methodological contribution by repurposing validated GDR indicators, traditionally used for dietary assessment, to evaluate the quality of retail food environments, offering a robust tool to inform interventions aimed at reducing nutrition-related NCDs. By quantitatively capturing both healthy and unhealthy food groups, this approach provides a systematic and replicable approach for monitoring and tracking changes in retail food environment quality over time. The indicators also establish a framework for cross-context comparisons, enabling the evaluation of food environments across diverse geographic and socioeconomic settings.

Furthermore, the integration of spatial autocorrelation offers an innovative dimension for characterizing food environments, highlighting clustering patterns and identifying hot spots and cold spots of healthy and unhealthy food availability. These spatial insights generate locally relevant evidence that can guide policymakers in designing targeted, context-specific strategies. Together, the combined use of retail food environment indices and spatial analysis provides a comprehensive framework for understanding and improving food environments. By bridging methodological innovation with policy relevance, this study advances the capacity to monitor retail food environments and supports the transformation of food systems toward healthier and more equitable outcomes.

## Author contributions

The authors’ responsibilities were as follows – NOO: conceptualization, methodology, investigation, formal analysis, writing-original draft, visualization; THA: software, formal analysis, writing-review and editing; IJ, CT: funding acquisition, writing-review and editing; FSUB: writing-review and editing; JK, RT: supervision, writing-review and editing; and all authors: read and approved the final manuscript.

## Data availability

The dataset underpinning the findings of this study has been published and is openly accessible through Harvard Dataverse https://hdl.handle.net/10568/177340 or https://doi.org/10.7910/DVN/YPVRMJ.

## Funding

We would like to thank all funders who supported this research through their contributions to the the Consultative Group on International Agricultural Research (CGIAR) Trust Fund (https://www.cgiar.org/funders/) and the Better Diets and Nutrition Science Program (https://www.cgiar.org/cgiar-research-porfolio-2025-2030/better-diets-and-nutrition/), as well as the Healthy Diet for Africa Project (HD4A), which is funded by the European Union (grant number: 01083388 - HORIZON-CL6-2022-FARM2FORK-01) and Biovision Foundation Switzerland (grant number: BV DPH_011). However, the views and opinions expressed are those of the author(s) only and do not necessarily reflect those of the European Union or the European Research Executive Agency. Neither the European Union nor the granting authority can be held responsible for them.

## Declaration of generative AI and AI-assisted technologies in the writing process

During the preparation of this manuscript, the author(s) used ChatGPT-4o for the purpose of editing text. After using this tool/service, the author(s) reviewed and edited the content as needed and take(s) full responsibility for the content of the publication.

## Conflict of interest

The authors report no conflicts of interest.
