# Supplementary material for: Adapting Global Dietary Recommendation Indices to Assess Retail Food Environment Quality: Spatial Insights from Rural and Urban Kenya
Source: Curr Dev Nutr. 2026 Apr 3;10(5):107685. doi: 10.1016/j.cdnut.2026.107685 (PMC13158746; doi:10.1016/j.cdnut.2026.107685)
Supplement: Multimedia component 1 [file mmc1.docx]

**SUPPLEMENTARY MATERIALS**

**Supplementary Material A: Description of the different retail outlet types**

| **Retail type** | **Retail description** |
| --- | --- |
| Butchers | Sell animal source food, especially flesh. May offer cutting of meat to pieces for customers on request |
| Cereal shop | Operates in permanent or semi-permanent structures. Sell in small quantities based on customers' demand. Mostly owned by individuals. |
| Cooked food street retailers | Small temporary roadside stands. Offer cooked foods. Most are set up daily, and operating time is based on the food sold individually. |
| Farm-gate sale | Offers fresh produce at the farm gate. Mostly family owned |
| Home-based retailers | Operates within home compound, mainly in a rural set-up. Offer a variety of products from fresh to processed foods to non-food items. Mostly operate in semi-permanent structures |
| Kiosk | Offer over-the-counter service with a minimal variety of foods. Sell in small quantities. Offer both food and non-food items. They are mostly individually owned. Operates mostly in semi-permanent structures |
| Mobile retailers | No fixed locations; move around on the streets and in residential areas. Offer a single or a few food varieties. |
| Modern restaurant | Offers cooked, ready-to-eat meals and/or dishes. Operates in permanent or semi-permanent structures. Customers enter the structure and sit to eat. May also offer takeaway |
| Mon-and-pop shop | Offer over-the-counter service. Sell in small quantities. Offer both food and non-food items that are mostly individually owned. Operates in permanent structures |
| Stalls/Tabletop retailers | Operate in temporary stands. Shred vegetables for customers on request. Are mostly individually owned |
| Supermarket | Offer self-service and have a large variety of foods and brands. Offers highly processed foods. Sell both food and non-food products. Operate mostly in permanent structures. |
| Wholesalers | Sell food products in bulk to retailers or directly to customers. Offer both food and non-food items. Offers lower prices. Operate in permanent structures. |

Table adapted from (33)

**Supplementary Material B**

**Fixed regression on the association between retail food environment indices at 50 m, 100 m and 200 m retail neighborhoods and retail food environment characteristics**

| **Food environment quality at 50 m retail neighborhood (N=2086)** | | | | | | | | | | | |
| --- | --- | --- | --- | --- | --- | --- | --- | --- | --- | --- | --- |
| **Characteristic** | **HFES** | | |  | **UFES** | | |  | **FEQI** | | |
|  | **IRR** | **95% CI** | **p-value** |  | **IRR** | **95% CI** | **p-value** |  | **IRR** | **95% CI** | **p-value** |
| **Retail location** |  |  |  |  |  |  |  |  |  |  |  |
| Rural | Reference | — |  |  | — | — |  |  | — | — |  |
| Urban | 1.08 | 1.05, 1.12 | **<0.001** |  | 1.01 | 0.98, 1.05 | 0.500 |  | 1.05 | 1.02, 1.08 | **<0.001** |
| **Retail food space** |  |  |  |  |  |  |  |  |  |  |  |
| 100% food space | Reference | — |  |  | — | — |  |  | — | — |  |
| Between 75% to 100% | 0.99 | 0.94, 1.03 | 0.600 |  | 1.00 | 0.95, 1.05 | >0.900 |  | 0.99 | 0.95, 1.03 | 0.600 |
| Between 75% to 50% | 0.98 | 0.92, 1.05 | 0.600 |  | 1.03 | 0.96, 1.10 | 0.400 |  | 0.97 | 0.92, 1.03 | 0.300 |
| Between 50% to 25% | 0.99 | 0.91, 1.09 | >0.900 |  | 1.01 | 0.92, 1.12 | 0.800 |  | 0.99 | 0.91, 1.07 | 0.800 |
| Below 25% | 0.88 | 0.76, 1.01 | 0.076 |  | 0.91 | 0.78, 1.06 | 0.200 |  | 0.96 | 0.85, 1.08 | 0.500 |
| **Gender** |  |  |  |  |  |  |  |  |  |  |  |
| Female | Reference | — |  |  | — | — |  |  | — | — |  |
| Male | 1.03 | 0.99, 1.07 | 0.200 |  | 1.03 | 0.98, 1.07 | 0.200 |  | 1.00 | 0.97, 1.04 | >0.900 |
| Mixed gender | 0.99 | 0.88, 1.11 | 0.900 |  | 1.03 | 0.91, 1.16 | 0.600 |  | 0.97 | 0.88, 1.07 | 0.600 |
| Multiple females | 0.89 | 0.73, 1.06 | 0.200 |  | 0.95 | 0.78, 1.15 | 0.600 |  | 0.95 | 0.81, 1.10 | 0.500 |
| Multiple males | 1.18 | 0.92, 1.49 | 0.200 |  | 1.06 | 0.80, 1.38 | 0.700 |  | 1.09 | 0.88, 1.34 | 0.400 |
| **Retail typology** |  |  |  |  |  |  |  |  |  |  |  |
| Mobile retail | Reference | — |  |  | — | — |  |  | — | — |  |
| Butcher | 1.06 | 0.96, 1.18 | 0.200 |  | 1.08 | 0.96, 1.20 | 0.200 |  | 1.00 | 0.91, 1.09 | >0.900 |
| Cereal shop | 1.12 | 0.98, 1.28 | 0.100 |  | 0.98 | 0.84, 1.13 | 0.800 |  | 1.10 | 0.98, 1.23 | 0.100 |
| Cooked food street vendor | 1.07 | 1.00, 1.15 | 0.065 |  | 1.02 | 0.95, 1.10 | 0.600 |  | 1.04 | 0.98, 1.10 | 0.200 |
| Farm gate sale | 0.36 | 0.25, 0.50 | **<0.001** |  | 0.07 | 0.03, 0.15 | **<0.001** |  | 1.15 | 0.96, 1.36 | 0.120 |
| Home retail | 0.57 | 0.42, 0.76 | **<0.001** |  | 0.70 | 0.52, 0.92 | **0.013** |  | 0.89 | 0.72, 1.08 | 0.200 |
| Kiosk | 1.02 | 0.95, 1.11 | 0.600 |  | 1.03 | 0.95, 1.12 | 0.500 |  | 1.00 | 0.94, 1.07 | >0.900 |
| Supermarket | 0.94 | 0.65, 1.32 | 0.700 |  | 1.15 | 0.81, 1.59 | 0.400 |  | 0.86 | 0.62, 1.16 | 0.300 |
| Mom-and-pop shops | 1.31 | 0.92, 1.80 | 0.120 |  | 1.10 | 0.73, 1.58 | 0.600 |  | 1.16 | 0.84, 1.54 | 0.300 |
| Modern restaurant | 1.05 | 0.87, 1.26 | 0.600 |  | 1.06 | 0.87, 1.28 | 0.600 |  | 1.00 | 0.85, 1.17 | >0.900 |
| Stalls/Tabletop | 1.17 | 1.09, 1.25 | **<0.001** |  | 1.05 | 0.97, 1.13 | 0.200 |  | 1.09 | 1.02, 1.15 | **0.007** |
| Wholesalers | 1.12 | 0.97, 1.29 | 0.130 |  | 1.05 | 0.90, 1.22 | 0.500 |  | 1.05 | 0.93, 1.19 | 0.400 |
| **Model fit** |  |  |  |  |  |  |  |  |  |  |  |
| PseudoR^2^ | 0.017 |  |  |  | 0.016 |  |  |  | 0.006 |  |  |
| AIC | 9283.2 |  |  |  | 8520.4 |  |  |  | 9285.3 |  |  |
| **Food environment quality at 100m retail neighbourhood (N=2086)** | | | | | | | | | | | |
| **Characteristic** | **HFES** | | |  | **UFES** | | |  | **FEQI** | | |
|  | **IRR** | **95% CI** | **p-value** |  | **IRR** | **95% CI** | **p-value** |  | **IRR** | **95% CI** | **p-value** |
| **Retail location** |  |  |  |  |  |  |  |  |  |  |  |
| Rural | Reference | — |  |  | — | — |  |  | — | — |  |
| Urban | 1.09 | 1.05, 1.12 | **<0.001** |  | 1.01 | 0.97, 1.04 | 0.600 |  | 1.06 | 1.03, 1.09 | **<0.001** |
| **Retail food space** |  |  |  |  |  |  |  |  |  |  |  |
| 100% food space | Reference | — |  |  | — | — |  |  | — | — |  |
| Between 75% to 100% | 1.00 | 0.96, 1.04 | 0.900 |  | 1.00 | 0.95, 1.05 | >0.900 |  | 1.00 | 0.96, 1.04 | >0.900 |
| Between 75% to 50% | 0.98 | 0.92, 1.04 | 0.500 |  | 1.01 | 0.94, 1.07 | 0.800 |  | 0.98 | 0.93, 1.03 | 0.400 |
| Between 50% to 25% | 0.99 | 0.91, 1.08 | 0.900 |  | 1.01 | 0.92, 1.11 | 0.800 |  | 0.99 | 0.91, 1.06 | 0.700 |
| Below 25% | 0.98 | 0.86, 1.12 | 0.800 |  | 0.98 | 0.84, 1.13 | 0.800 |  | 1.00 | 0.89, 1.12 | >0.900 |
| **Gender** |  |  |  |  |  |  |  |  |  |  |  |
| Female | Reference | — |  |  | — | — |  |  | — | — |  |
| Male | 1.01 | 0.98, 1.05 | 0.500 |  | 1.01 | 0.97, 1.06 | 0.500 |  | 1.00 | 0.97, 1.03 | >0.900 |
| mixed gender | 0.97 | 0.87, 1.08 | 0.600 |  | 1.00 | 0.89, 1.12 | >0.900 |  | 0.98 | 0.89, 1.07 | 0.600 |
| Multiple females | 0.97 | 0.82, 1.14 | 0.700 |  | 0.97 | 0.81, 1.17 | 0.800 |  | 0.99 | 0.85, 1.15 | >0.900 |
| Multiple males | 1.05 | 0.83, 1.32 | 0.700 |  | 1.01 | 0.77, 1.30 | >0.900 |  | 1.04 | 0.83, 1.27 | 0.700 |
| **Retail typology** |  |  |  |  |  |  |  |  |  |  |  |
| Mobile retail | Reference | — |  |  | — | — |  |  | — | — |  |
| Butcher | 0.99 | 0.90, 1.08 | 0.800 |  | 1.02 | 0.92, 1.13 | 0.700 |  | 0.98 | 0.90, 1.06 | 0.600 |
| Cereal shop | 1.03 | 0.91, 1.17 | 0.600 |  | 1.02 | 0.88, 1.17 | 0.800 |  | 1.01 | 0.90, 1.13 | 0.900 |
| Cooked food street vendor | 1.00 | 0.93, 1.06 | 0.900 |  | 1.02 | 0.95, 1.09 | 0.600 |  | 0.99 | 0.93, 1.04 | 0.600 |
| Farm gate sale | 0.35 | 0.25, 0.48 | **<0.001** |  | 0.14 | 0.08, 0.24 | **<0.001** |  | 1.06 | 0.89, 1.26 | 0.500 |
| Home retail | 0.54 | 0.41, 0.71 | **<0.001** |  | 0.68 | 0.51, 0.88 | **0.004** |  | 0.85 | 0.69, 1.03 | 0.110 |
| Kiosk | 0.96 | 0.90, 1.04 | 0.300 |  | 0.99 | 0.92, 1.07 | 0.800 |  | 0.98 | 0.92, 1.04 | 0.500 |
| Supermarket | 1.07 | 0.78, 1.43 | 0.700 |  | 1.07 | 0.75, 1.47 | 0.700 |  | 1.01 | 0.76, 1.32 | >0.900 |
| Mom-and-pop shops | 1.07 | 0.75, 1.47 | 0.700 |  | 1.02 | 0.68, 1.46 | >0.900 |  | 1.05 | 0.76, 1.40 | 0.800 |
| Modern restaurant | 0.99 | 0.83, 1.17 | >0.900 |  | 1.04 | 0.86, 1.24 | 0.700 |  | 0.97 | 0.83, 1.12 | 0.700 |
| Stalls/Tabletop | 1.04 | 0.97, 1.11 | 0.300 |  | 1.02 | 0.95, 1.10 | 0.500 |  | 1.01 | 0.96, 1.08 | 0.600 |
| Wholesalers | 1.04 | 0.91, 1.19 | 0.500 |  | 1.02 | 0.88, 1.19 | 0.700 |  | 1.02 | 0.90, 1.15 | 0.800 |
| **Model fit** |  |  |  |  |  |  |  |  |  |  |  |
| PseudoR^2^ | 0.016 |  |  |  | 0.014 |  |  |  | 0.004 |  |  |
| AIC | 8888.3 |  |  |  | 8336.5 |  |  |  | 9061.6 |  |  |
| **Food environment quality 200m retail neighbourhood (N=2086)** | | | | | | | | | | | |
| **Characteristic** | **HFES** | | |  | **UFES** | | |  | **FEQI** | | |
|  | **IRR** | **95% CI** | **p-value** |  | **IRR** | **95% CI** | **p-value** |  | **IRR** | **95% CI** | **p-value** |
| **Retail location** |  |  |  |  |  |  |  |  |  |  |  |
| Rural | Reference | — |  |  | — | — |  |  | — | — |  |
| Urban | 1.08 | 1.05, 1.11 | **<0.001** |  | 1.01 | 0.98, 1.05 | 0.500 |  | 1.06 | 1.03, 1.09 | **<0.001** |
| **Retail food space** |  |  |  |  |  |  |  |  |  |  |  |
| 100% food space | Reference | — |  |  | — | — |  |  | — | — |  |
| Between 75% to 100% | 1.00 | 0.96, 1.04 | >0.900 |  | 1.00 | 0.96, 1.05 | >0.900 |  | 1.00 | 0.96, 1.04 | >0.900 |
| Between 75% to 50% | 0.98 | 0.93, 1.04 | 0.500 |  | 1.01 | 0.94, 1.07 | 0.800 |  | 0.98 | 0.93, 1.03 | 0.500 |
| Between 50% to 25% | 1.01 | 0.93, 1.09 | 0.900 |  | 1.03 | 0.94, 1.13 | 0.500 |  | 0.98 | 0.91, 1.06 | 0.700 |
| Below 25% | 1.00 | 0.88, 1.14 | >0.900 |  | 1.00 | 0.87, 1.15 | >0.900 |  | 1.00 | 0.89, 1.12 | >0.900 |
| **Gender** |  |  |  |  |  |  |  |  |  |  |  |
| Female | Reference | — |  |  | — | — |  |  | — | — |  |
| Male | 1.01 | 0.98, 1.05 | 0.500 |  | 1.01 | 0.97, 1.05 | 0.700 |  | 1.01 | 0.97, 1.04 | 0.800 |
| mixed gender | 0.98 | 0.88, 1.09 | 0.800 |  | 1.02 | 0.91, 1.13 | 0.800 |  | 0.98 | 0.89, 1.07 | 0.600 |
| Multiple females | 0.99 | 0.84, 1.16 | >0.900 |  | 1.01 | 0.84, 1.19 | >0.900 |  | 0.99 | 0.85, 1.15 | >0.900 |
| Multiple males | 1.04 | 0.82, 1.30 | 0.700 |  | 1.00 | 0.76, 1.28 | >0.900 |  | 1.03 | 0.83, 1.27 | 0.800 |
| **Retail typology** |  |  |  |  |  |  |  |  |  |  |  |
| Mobile retail | Reference | — |  |  | — | — |  |  | — | — |  |
| Butcher | 0.98 | 0.90, 1.08 | 0.800 |  | 1.00 | 0.90, 1.11 | >0.900 |  | 0.99 | 0.90, 1.07 | 0.800 |
| Cereal shop | 1.02 | 0.90, 1.16 | 0.700 |  | 1.03 | 0.89, 1.17 | 0.700 |  | 1.00 | 0.89, 1.12 | >0.900 |
| Cooked food street vendor | 1.00 | 0.94, 1.06 | >0.900 |  | 1.01 | 0.95, 1.09 | 0.700 |  | 0.99 | 0.93, 1.05 | 0.700 |
| Farm gate sale | 0.41 | 0.30, 0.55 | **<0.001** |  | 0.21 | 0.13, 0.32 | **<0.001** |  | 1.07 | 0.90, 1.27 | 0.400 |
| Home vendor | 0.58 | 0.44, 0.74 | **<0.001** |  | 0.68 | 0.52, 0.88 | **0.004** |  | 0.87 | 0.71, 1.06 | 0.200 |
| Kiosk | 0.97 | 0.91, 1.04 | 0.500 |  | 0.99 | 0.92, 1.07 | 0.800 |  | 0.99 | 0.93, 1.05 | 0.700 |
| Supermarket | 1.06 | 0.78, 1.42 | 0.700 |  | 1.08 | 0.77, 1.47 | 0.600 |  | 1.00 | 0.74, 1.31 | >0.900 |
| Mom-and-pop shops | 1.04 | 0.73, 1.44 | 0.800 |  | 1.11 | 0.76, 1.56 | 0.600 |  | 0.96 | 0.69, 1.30 | 0.800 |
| Modern restaurant | 1.00 | 0.84, 1.18 | >0.900 |  | 1.04 | 0.86, 1.23 | 0.700 |  | 0.97 | 0.83, 1.13 | 0.700 |
| Stalls/Tabletop | 1.03 | 0.96, 1.10 | 0.400 |  | 1.02 | 0.95, 1.10 | 0.500 |  | 1.01 | 0.95, 1.07 | 0.800 |
| Wholesalers | 1.03 | 0.90, 1.17 | 0.700 |  | 1.01 | 0.87, 1.17 | 0.800 |  | 1.01 | 0.89, 1.14 | 0.800 |
| **Model fit** |  |  |  |  |  |  |  |  |  |  |  |
| PseudoR^2^ | 0.013 |  |  |  | 0.012 |  |  |  | 0.003 |  |  |
| AIC | 8798.4 |  |  |  | 8395.9 |  |  |  | 8998.4 |  |  |

RHFES=Retail Healthy Food Environment Score; RUFES=Retail Unhealthy Food Environment Score; RFEQI=Retail Healthy Food Environment Quality Index; CI=Confidence Interval; Retail food space is the proportion of the outlet space occupied by food; Sex indicates the sex of the person selling food at the retail outlet; Both sexes indicates that the retail outlet had both male and female retailers at the outlet; Multiple females indicates that the outlet has more than one female; and Multiple males indicates that the outlet had more than one male.
